# Enzyme Sequestration as a Tuning Point in Controlling Response Dynamics of Signalling Networks

**DOI:** 10.1371/journal.pcbi.1004918

**Published:** 2016-05-10

**Authors:** Song Feng, Julien F. Ollivier, Orkun S. Soyer

**Affiliations:** 1 School of Life Sciences, University of Warwick, Coventry, United Kingdom; 2 Biojazz Research, Montreal, Quebec, Canada; ETH Zurich, SWITZERLAND

## Abstract

Signalling networks result from combinatorial interactions among many enzymes and scaffolding proteins. These complex systems generate response dynamics that are often essential for correct decision-making in cells. Uncovering biochemical design principles that underpin such response dynamics is a prerequisite to understand evolved signalling networks and to design synthetic ones. Here, we use *in silico* evolution to explore the possible biochemical design space for signalling networks displaying ultrasensitive and adaptive response dynamics. By running evolutionary simulations mimicking different biochemical scenarios, we find that enzyme sequestration emerges as a key mechanism for enabling such dynamics. Inspired by these findings, and to test the role of sequestration, we design a generic, minimalist model of a signalling cycle, featuring two enzymes and a single scaffolding protein. We show that this simple system is capable of displaying both ultrasensitive and adaptive response dynamics. Furthermore, we find that tuning the concentration or kinetics of the sequestering protein can shift system dynamics between these two response types. These empirical results suggest that enzyme sequestration through scaffolding proteins is exploited by evolution to generate diverse response dynamics in signalling networks and could provide an engineering point in synthetic biology applications.

## Author Summary

Biological systems utilise signalling networks that are composed of multiple interacting proteins to process environmental information. The function of these networks is critical for cells to respond and adapt to their environment by converting environmental signals to appropriate cellular response dynamics. As results of evolution, these signalling networks display certain evolutionary design principles (i.e. common structural and dynamical features) that allow them to implement specific functions. Here, we use an *in silico* evolution approach to simulate the emergence of signalling networks that are capable of two specific types of response dynamics: switch-like and/or adaptive response dynamics. These two response dynamics underpin cellular decision-making and homeostasis. By analysing the evolved networks, we discover that enzyme sequestration is a key feature involved in achieving both types of response dynamics. Based on this finding, we design a minimalistic signalling motif featuring enzyme sequestration through a scaffold protein. We demonstrate that this motif can achieve both response dynamics and furthermore the type of response can be controlled through the concentration level of the scaffold protein. These results highlight enzyme sequestration as a potential evolutionary design principle to achieve key response dynamics in natural signalling networks and as an engineering route in synthetic biology.

## Introduction

Molecular signalling networks enable cells to generate appropriate dynamical responses to external signals including pulsed, oscillatory, ultrasensitive, and adaptive dynamics [1,2] [3]. Such response dynamics are also implemented in human-engineered systems, motivating the use engineering principles to understand and engineer cellular networks [3,4]. This approach has been particularly useful in the context of gene regulatory networks, where feedback and feedforward control are successfully used to explain and even engineer specific response dynamics [5–12]. While these studies demonstrate the usefulness of engineering principles, particularly feedback control, in understanding and modulating biological systems [3], there is also great interest to discover and understand potential design principles that are unique to cellular networks and that are exploited by evolution to generate specific system dynamics [13] [14,15].

One way to identify potential evolutionary design principles is to look for features conserved across different cellular systems. For example, the high prevalence of phosphorylation-dephosphorylation cycles in signalling networks and of branching points in metabolic networks led to their identification as potential mediators of ultrasensitive dynamics [16,17]. Similarly, several common biochemical features of signalling networks were identified as mediators of specific response dynamics: bifunctional enzymes mediating adaptive and pulse dynamics [18,19], multi-site phosphorylation mediating multistability [20–23], and phosphorelays mediating ultrasensitivity and multistability [24–28].

An alternative approach for identification of potential design principles in cellular networks is to use *in silico* evolution [13] [29] [30,31]. Through the mimicking of biological evolution of cellular networks in the computer, *in silico* evolution can generate many systems with a desired response. These systems can then be analysed to identify their key features mediating specific response dynamics. The application of this approach led to the identification and subsequent experimental implementation of sequestration as a mechanism for generating bistability and oscillation in gene regulatory networks [14,32,33] and also to uncovering the principle of adaptive sorting in ligand-receptor interactions, which is analogously featured in immune recognition [34]. These examples illustrate the potential utility of *in silico* evolution to discover subtle biochemical processes that could not be readily deduced from observations on network connectivity. In addition, the evolutionary approach allows exploring the impact of specific environmental and cellular conditions on the evolution of different design principles [35–40]. Given that many different potential design principles can give rise to a certain dynamical response, such insights could be useful for increasing our ability to predict which designs are more likely to be found under which ecological and evolutionary setting.

Motivated by this potential, we use here a recently developed *in silico* evolutionary simulation framework to explore the design principles of ultrasensitive and adaptive dynamics in signalling networks. Both response dynamics are prevalent in biological systems and can be formalised and quantified mathematically (S1 Fig, also see Methods). We show that enzyme sequestration emerges as a key mechanism for enabling ultrasensitivity. Interestingly, this same mechanism also emerges in networks selected for adaptive dynamics, and is implemented in such a way to mediate a contrasting effect on the activities of kinases and phosphatases. Based on these findings, we design a generic model of a minimalist signalling cycle motif, featuring a scaffolding protein. We show that enzyme sequestration in this motif enables it to generate both ultrasensitive and adaptive dynamics under biologically relevant parameter regimes. Furthermore, we show that the dynamics of such a motif can be tuned between adaptive and ultrasensitive responses through modulation of the concentration or kinetic parameters of the sequestrating protein. These findings indicate that enzyme sequestration through scaffolding proteins provides evolution with a means to generate systems with plastic response dynamics and that this design principle could be equally exploited in synthetic biology.

## Results

**Fig 1:**
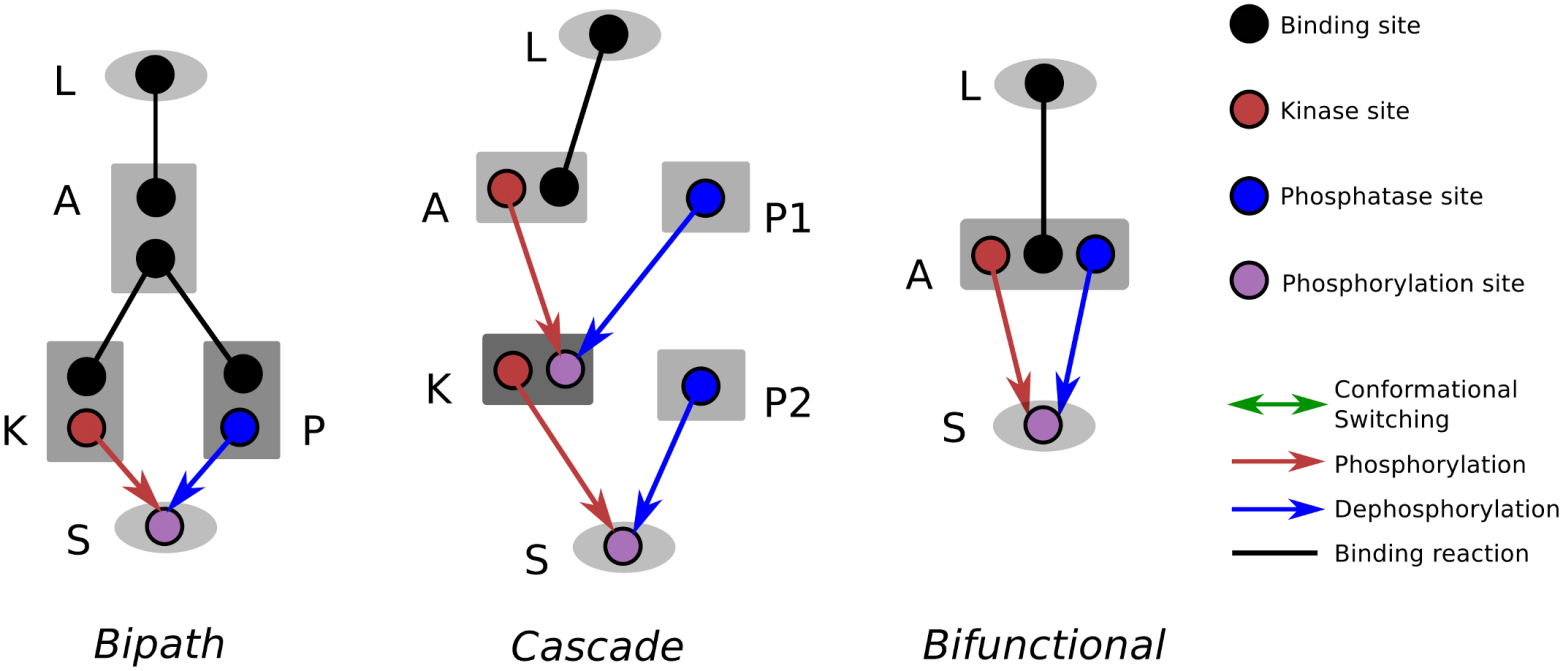
Starting network topologies in evolutionary simulations. Structure of three different *seed* networks labelled as *Bipath* (a signalling system featuring a branching point), *Cascade* (a simple, linear signalling system with phosphatases for each step), and *Bifunctional* (a signalling system featuring a bifunctional protein). The ligand and the output protein (e.g. a transcription factor) are shaped as oval, while all other signalling proteins (e.g. receptor/adaptor proteins, kinases, or phosphatases) are shaped as rectangle. Black line represents binding reaction between two sites. Red arrows represent phosphorylation reactions between a kinase site (red) and a phosphorylation site (purple). Blue arrows represent dephosphorylation reactions between a phosphatase site (blue) and a phosphorylation site.

To explore design principles for generating ultrasensitive and adaptive response dynamics in signalling networks, we used an *in silico* evolution platform that combines rule-based models of such networks with an evolutionary algorithm [41] (see Methods). Using this framework, we have evolved signalling networks under different cellular conditions and from three different starting networks composed of an input-receiving protein (*L*), an output protein (*S*), and proteins with binding, kinase and phosphatase activities and labelled as adaptor proteins (*A*), kinases (*K*) or phosphatases (*P*) (Fig 1). The initial structures were selected based on common observations from natural signalling networks. In particular, the “cascade” topology is based on the signalling cascades such as the Mitogen Activated Protein Kinase (MAPK) signalling networks [42,43]; the “bipath” topology is based on the observations that cells utilise different signalling pathways that share specific elements leading to “cross-talk”, as seen for example in the signalling pathways controlling yeast mating and filamentous growth responses [44–46]; the “bifunctional” topology is inspired by observations that many kinases can also display significant phosphatase activity, or can readily attain such activity via few mutations [47–51] Furthermore, this motif is selected as it provides a particularly minimal starting point for evolution, where we assume a “generalist” enzyme that contains both kinase and phosphatase activities initially and that can evolve these activities further via mutations and protein duplication. The cellular conditions were selected to mimic the presence or absence of enzyme saturation, which can mediate ultrasensitivity in signalling cycles [16] but might be lacking in natural systems [52,53]. Thus, the evolutionary simulations allowed us to explore the role of these different features. We used specific selection criteria that operate on the response dynamics resulting from the network in presence of a signal profile (see Methods and S1 Fig). On quantifying the response dynamics, the signal profile is implemented by perturbing concentration of the input protein (*L* in Fig 1), while the output response is defined as concentration of phosphorylated output protein (*S* in Fig 1). We ran 10 simulations for each of the conditions and for selecting ultrasensitive and adaptive responses, resulting in a total of 60 simulations for each scenario. Each of these simulations resulted in more complex networks evolving compared to the starting ones (see S1 Table and Supporting Information for evolved network models).

### *In silico* evolved ultrasensitive networks display saturation, sequestration, and allosteric regulation of enzymes acting on the output protein

It has been shown theoretically that a simple signalling motif comprising a kinase, a phosphatase, and their substrate can lead to an ultrasensitive input-response relation when the enzymes are fully saturated by their substrate [16]. This mechanism is termed zero-order sensitivity and can be achieved by having kinetic parameters that favour complex formation among enzymes and the substrates, and by having a large ratio of the total concentration of substrate to that of enzymes [16].

**Fig 2:**
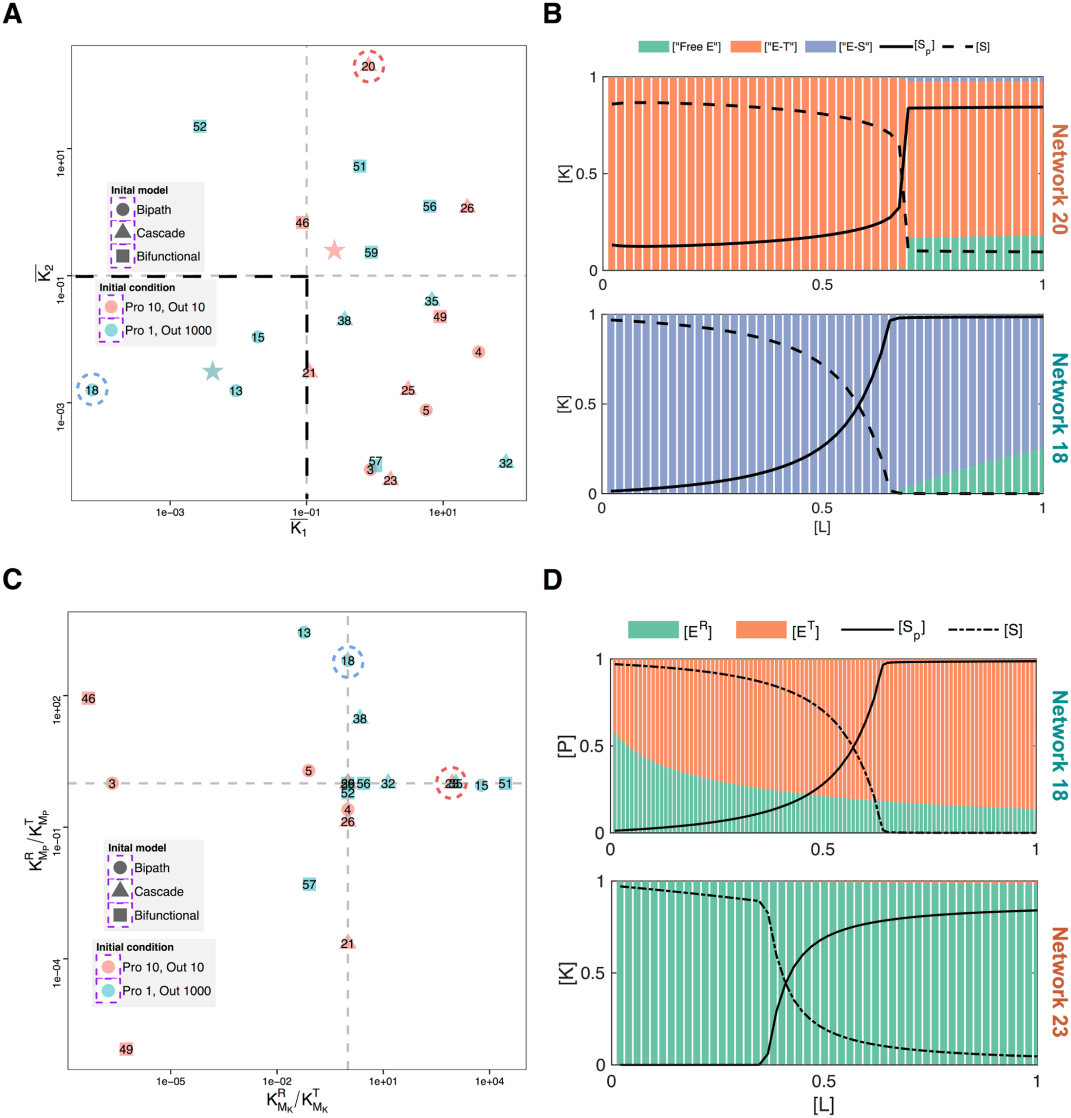
Analysis of evolved ultrasensitive networks. (A) Average saturation of enzymes in all of the evolved ultrasensitive networks. The average saturation of enzymes is calculated as the geometric mean of individual Michaelis-Menten constants of the different kinases and phosphatases and their allosteric states normalised by the substrate concentration (kinase, *K*_*1*_, or phosphatase, *K*_*2*_). The shape of each data point represents different starting structures to the evolutionary simulations (see S1 Fig). The colours of the data points represent two different evolutionary scenarios; blue: output protein [*S*_*total*_] = 1000, other signalling proteins (A*) concentrations [A*_total_] = 1; red: output protein [*S*_*total*_] = 10, other signalling proteins concentrations [A*_total_] = 10. The blue and red, star-shaped points indicate the average value of the enzyme saturation resulting from these initial concentrations at the start of the evolutionary simulations. Each data point is further labelled with the unique identification number used for each evolutionary simulation. (B) The fraction of different forms of the kinase (*y*-axis) against the ligand concentration (*x*-axis) for two different evolved networks (network 20 and 18 in S2 Fig). The fractions of the different forms of the kinase are the substrate-accessible (green), substrate-inaccessible (orange), and substrate-bound (blue) forms. This data is overlaid with the dose-response dynamics; the solid and dashed lines show the steady state concentration of phosphorylated (i.e. response) and unphosphorylated substrate respectively at a given input level. (C) Ratio between *K*_*M*_ values of different conformational states (relaxed “R” state and tensioned “T” states) for kinase (*x*-axis) and phosphatase (*y*-axis). The colours, shapes and numbers on the dots are the same as in (A). For enzymes without allosteric regulation the ratio are set to one, so that there are no distinctive conformational differences. (D) The fraction of different forms of the phosphatase (top) and kinase (bottom) (*y*-axis) against the ligand concentration (*x*-axis) for two different evolved networks (network 18 and 23 in S2 Fig). The different forms of the enzymes are the different conformational states, relaxed “R” state (green) and tensioned “T” state (orange). These are overlaid with dose-response dynamics; the solid and dashed lines show the steady state concentration of phosphorylated (i.e. response) and unphosphorylated substrate respectively.

We found that when conditions allow, zero-order sensitivity evolves *in silico*. Of the 30 simulations, which were started with a high ratio of output protein to signalling protein concentrations, 11 were successful and have resulted in the emergence of ultrasensitivity (Fig 2A, blue points). Of these, 3 simulations resulted in kinetic parameters where both kinases and phosphatases that are directly acting on the output protein were saturated (the lower, left quadrant in Fig 2A). These results confirm that the *in silico* simulation framework can recover a known biochemical mechanism—enzyme saturation by substrate—for achieving ultrasensitivity.

Interestingly, however, 8 of the successful simulations have resulted in enzyme kinetic parameters evolving out of the highly saturated regime (note that we use the word “enzyme” here and in the rest of this section, only in reference to evolved proteins that have catalytic activity towards the output protein). This observation is interesting because enzyme saturation mediated zero-order sensitivity might not be relevant for many biological systems where the ratio of substrate to enzyme concentrations is found to be low [52,53]. To further explore evolution of ultrasensitivity under such non-saturating conditions, we ran evolutionary simulations with equal starting concentrations for the substrate and signalling proteins. Although concentration of signalling proteins could freely evolve in these simulations, enzyme saturation was expected to be difficult to evolve, further favouring the evolution of alternative mechanisms for ultrasensitivity. Indeed, none of the emerging ultrasensitive networks from these simulations (10 out of 30 simulations) display strong enzyme saturation (Fig 2A, red points). Together with the ultrasensitive networks that started with high concentrations of the substrate, but did not evolve enzyme saturation, these ultrasensitive networks clearly utilize mechanisms other than enzyme saturation.

Analysing these networks (S2 Fig), we did not find any distinct structural features. However, we found that in many evolved networks with parameters in the non-saturating regime, there is a high prevalence of enzyme sequestration (Fig 2B) and also allosteric regulation of enzyme activity (Fig 2C) by other signalling proteins. In theory, allosteric regulation of enzyme activity by upstream proteins that are activated by signals could implement a form of ultrasensitivity that could relax the need for enzyme saturation [54–56]. However, we found that for at least some networks, the ratio of allosteric forms of the enzymes barely changes across the input range (Fig 2D), showing that allosteric regulation is not the main or sole process enabling ultrasensitivity. This prompted us to analyse all of the evolved networks with regard to the prevalence of the different enzyme complexes. In particular, we calculated the average proportions of complexes formed between enzymes and the output protein *S* (notated as *ES* complexes), and complexes formed between other proteins (i.e. those that don’t have catalytic activity towards the output protein) and enzymes (notated as *ET* complexes). Note that these proportions can be seen as the average level of enzyme sequestration in the signalling network. This analysis revealed that most of the ultrasensitive networks evolved parameters that resulted in enzymes being bound in complexes i.e. they lie close to the line given by [*ET*] = 1 –[*ES*] (S3 Fig). Moreover, contrasting the results of evolutionary simulations where enzyme saturation was made difficult to evolve versus not, showed that the non-saturation scenario resulted in higher prevalence of *ET* complexes, i.e. enzyme being mostly titrated by other signalling proteins (see Fig 2A and S3 Fig). These results suggest that enzyme sequestration is a key mechanism enabling the evolution of ultrasensitivity. We analysed this proposition further below using a minimal model.

### Selection for adaptive dynamics leads to networks employing differential enzyme sequestration

**Fig 3:**
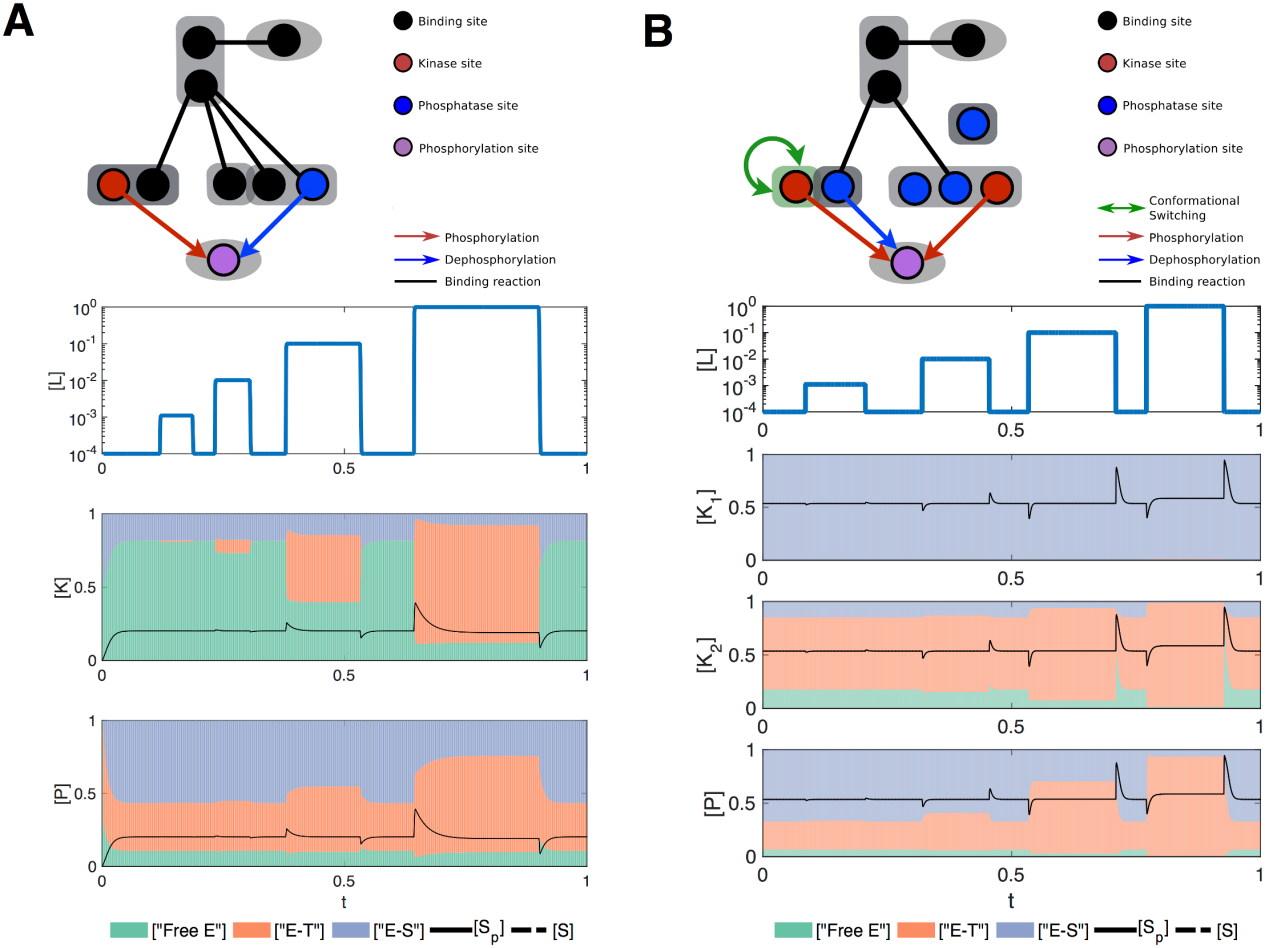
Analysis of evolved adaptive networks. (A) Structure and dynamics of the evolved adaptive network number 1. The upper panel shows a cartoon of the network. The oval shapes represent ligand (top) and the output protein (bottom) (e.g. substrate with a phosphorylation site, *S*), while rectangles represent all other signalling proteins (e.g. receptors, scaffolds, kinases, or phosphatases). Black lines represent the binding reaction between two sites. Red arrows represent a kinase site (red) phosphorylating a substrate phosphorylation site (purple). Blue arrows represent a phosphatase site (blue) dephosphorylating a substrate site. The green rectangle with a self-pointing green arrow indicates a protein domain whose conformational switching is allosterically regulated. The lower panels show the temporal dynamics of the input ([*L*], top) and the output protein (black lines in lower panels). The dynamics of the output protein is overlaid with the distribution of the different kinase ([*K*], middle panels) and phosphatase ([*P*], bottom panels) complexes: blue for enzyme-substrate complexes, green for free form of the enzymes that are accessible by the substrate, and red for complexes of enzymes with other signalling proteins. (B) Structure and dynamics of the evolved adaptive network number 2. Panels are as in (A).

To select networks with adaptive response dynamics, we implemented a stringent fitness function that required adaptation to input signals of several different magnitudes (Methods). We found only few of the evolutionary simulations resulting in networks with adaptive dynamics (2 out of 60 simulations), potentially due to the strictness of this fitness function. Interestingly, in both of these simulations, the final evolved networks contained a protein, the role of which implements a differential sequestration of the enzymes, e.g. by sequestrating them through a different number of binding sites (Fig 3A). The imbalanced sequestration affinity of this protein towards kinases and phosphatases enables the system to provide an initial response to a change in signal but then move back to same equilibrium point (Fig 3B). With every signal step, the kinase is titrated much faster compared to the phosphatase leading to an initial response that then settles back to previous levels, when sequestration levels of the kinase and phosphatase equilibrate (Fig 3). When the sequestering protein is fully bound, and the sequestration effect cannot operate anymore, the level of adaptation to signal is hampered (see Fig 3B). Interestingly, we find that such proteins acting on both kinases and phosphatases as seen in evolved adaptive networks are also featured in evolved ultrasensitive networks (see network 4 and network 13 in S2 Fig).

### Scaffolding protein enables ultrasensitivity and adaptive dynamics in a single signalling cycle

**Fig 4:**
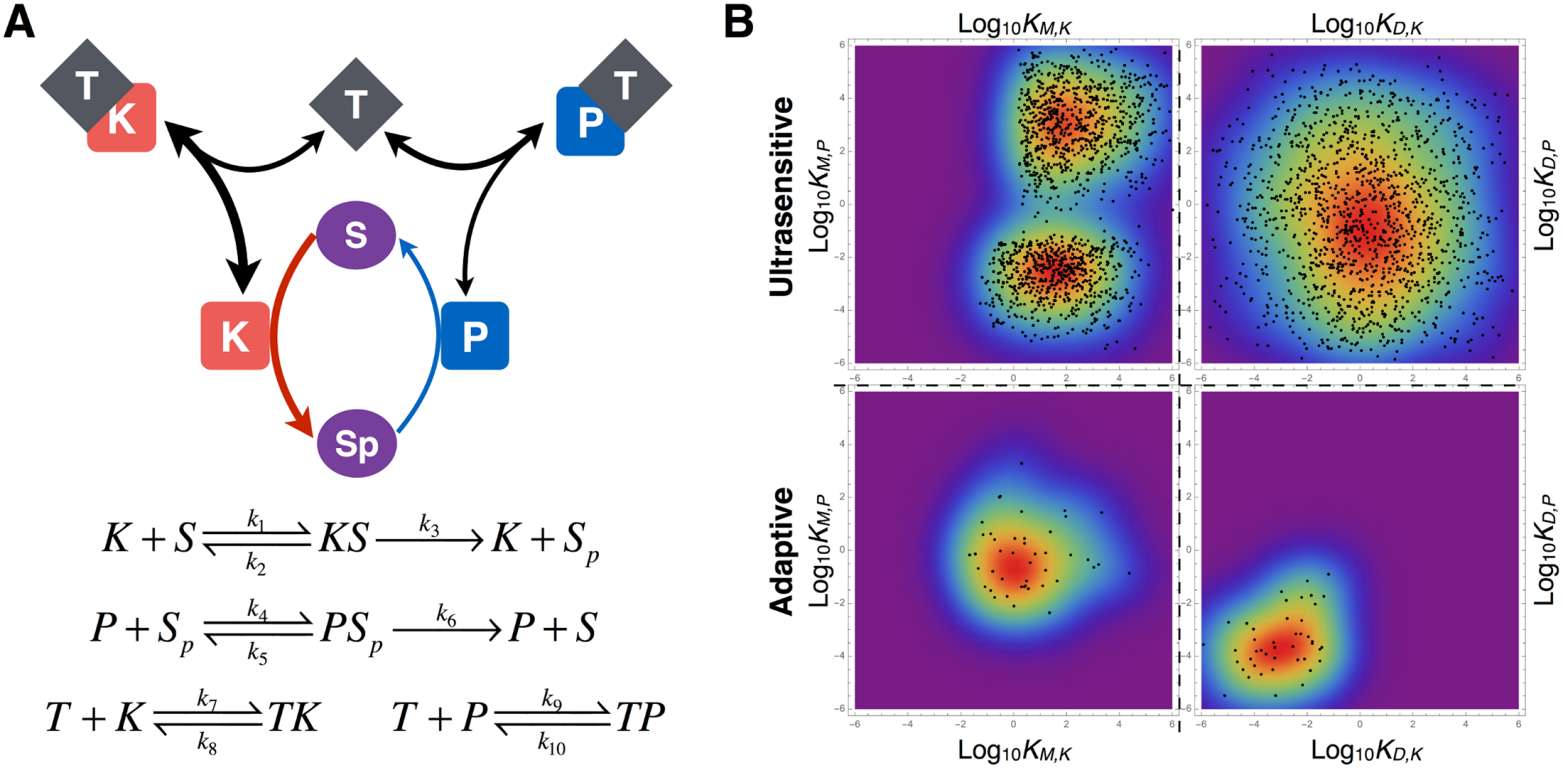
Designed signalling cycle motif and parameter space for adaptation and ultrasensitivity. (A) Cartoon showing the designed signalling cycle motif with a sequestering protein. The sequestrating protein (*T*) binds both the kinase (*K*) and phosphatase (*P*), which catalyse the phosphorylation and dephosphorylation of unphosphorylated (*S*) and phosphorylated (*S*_*p*_) substrate respectively. (B) The values of key parameters for achieving ultrasensitive (> 0.8) and adaptive (>0.3) responses, when assuming an enzyme-saturated regime ([*S*_*total*_] = 1, [*P*_*total*_] = 0.1). Panels on the left show the distribution of Michaelis-Menten constants, for kinase: *K*_*M*,*K*_ = (*k*_2_+*k*_3_)/*k*_1_ (*x*-axis) and phosphatase *K*_*M*,*P*_ = (*k*_5_+*k*_6_)/*k*_4_ (*y*-axis). Panels on the right show the distribution of affinities of sequestrating protein *T* with kinase and phosphatase: *K*_*D*,*K*_ = *k*_8_/*k*_7_ and *K*_*D*,*P*_ = *k*_10_/*k*_9_ Note that all four panels are plotted on the same logarithmic range. Each black dot represents a parameter set and the colours shows density of parameters.

The above findings suggest that scaffolding proteins that act in an enzyme sequestering capacity could allow implementation of both adaptive and ultrasensitive dynamics. Inspired by this observation and to test it, we developed a model of a simple signalling cycle motif that features enzyme sequestration, and where incoming signals are implemented as changes in kinase concentration (see Methods and Fig 4A). We analysed the ability of this model to generate ultrasensitive and adaptive responses by sampling 100,000 independent sets of kinetic parameters from a biologically feasible regime (Table 1). We find that this generic model can achieve both adaptive and ultrasensitive dynamics, whether we impose enzyme-saturating conditions or not (S4 Fig).

**Table 1:** Parameter ranges used for the sampling of signalling cycle model.

Parameters	*In silico*	Measure	Citation
Concentration (*μM*)	[10^−4^, 10]	[0.002, 1.8]	[57–63]
Phosphorylation (*s*^−1^)	[10^−3^, 10^3^]	[0.17, 8.87]	[57,59–61]
Dephosphorylation (*s*^−1^)	[10^−3^, 10^3^]	[0.06, 5.31]	[57,59–61]
Protein association (*μM*^−1^·*s*^−1^)	[10^−3^, 10^3^]	[0.10, 7.53]	[57,59–61]
Protein disassociation (*s*^−1^)	[10^−3^, 10^3^]	[0.015, 2.86]	[57,59–61]

For the case of ultrasensitive dynamics, analysis of all “successful” parameter sets showed two distinct parameter regimes leading to ultrasensitive dynamics (Fig 4B). These regimes relate to enzyme saturation (i.e. large or small *K*_*M*_ values); in one regime, the phosphatase has high affinity for the substrate and is fully saturated by it (small *K*_*M*,*P*_), while the kinase has high affinity for the sequestrating protein (S5C Fig). In the second parameter regime, both the kinase and the phosphatase have large *K*_*M*_ values indicating a lower affinity for the substrate. Thus, the enzymes are mainly bound to the sequestrating protein (small *K*_*D*_ values) and are in competition for that protein (S5C Fig). In both parameter regimes, small increases of incoming signals (i.e. small increase in kinase concentration) can be “absorbed” by increased sequestration of the kinase, while higher signal levels saturate this sequestration-mediated effect, resulting in significant amounts of free kinase in turn causing a switch to high phosphorylation rates. The difference between the two parameter regimes is that in the second regime, competition between the kinase and phosphatase for the sequestrating protein results in an additional feedback, where increased kinase levels enhance free phosphatase levels (through release from the sequestering protein). As expected from this analysis, we find that ultrasensitivity can only be generated in the second parameter regime (i.e. large *K*_*M*_ values and small *K*_*D*_ values) when we sample parameters by forcing either enzyme to be unsaturated by the substrate (S6 Fig).

In the case of adaptive dynamics, we find that the parameter regime leading to highly adaptive networks corresponds to competition between the kinase and phosphatase for the sequestering protein (i.e. small *K*_*D*_ values) (Figs 4B and S6). In this case, incoming signals temporarily bias this competition towards the free kinase, but subsequently, the kinase binds the sequestrating protein at the expense of the phosphatase. The resulting release of the phosphatase leads to the balancing of the phosphorylation and desphosphorylation rates, yielding adaptive dynamics (Fig 4B).

### Scaffolding protein can act as a tuning point to generate plastic response dynamics

The intriguing similarity of the mechanisms for adaptive and ultrasensitive dynamics suggests that there could be a parameter regime where the system could implement both dynamics. In particular, we note that there are parameter sets at the edges of the distinct regimes identified above and leading to ultrasensitive and adaptive dynamics (Fig 4B). Is it possible that such parameter sets result in system where response dynamics can be modulated by the sequestrating protein? In order to answer this question, we selected parameter sets from this boundary regime and sampled the concentration of sequestrating protein (*T*) while fixing all other parameters, aiming to check if simply varying the level of *T* could modulate the response dynamics.

**Fig 5:**
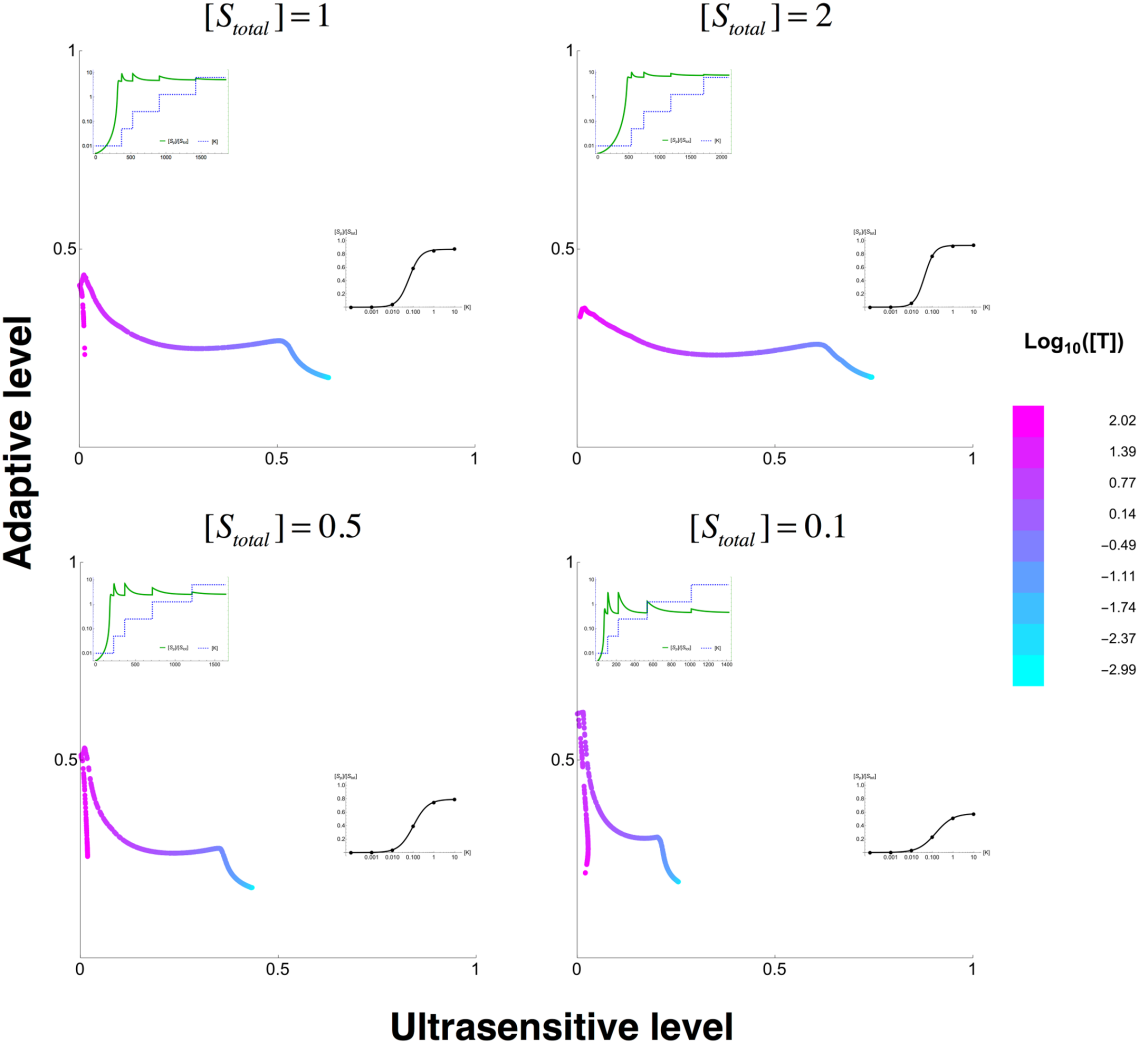
Modulation of response dynamics through tuning of scaffold protein concentration. The four panels show sampling the total concentration of scaffolding protein, [*T*_*total*_], when fixing all other parameters and with the total concentration of substrate [*S*_*total*_] as shown on the panels. The colour of each data point represents the concentration of the scaffolding protein concentration. In each panel, the best ultrasensitive or adaptive response dynamics that are achieved at a specific [*T*_*total*_] level are shown as insets. For adaptive responses (top left of each panel), the inset shows the concentration of the input ([*K*]) as a blue dashed line and the concentration of the output ([*S*_*p*_]) as a solid green line. For ultrasensitive dynamics (bottom right of each panel), the inset shows the steady state fraction of phosphorylated substrate ([*S*_*p*_]/[*S*_*tot*_]) at a given input level ([*K*]) as a black solid line.

A few systems did indeed show such modulation, behaving with adaptive or ultrasensitive dynamics at two distinctive concentrations of *T* (Fig 5). Interestingly, this modulation is influenced directly by the concentration of substrate *S*; at high (low) substrate concentration, modulation by *T* allows an extended shift towards ultrasensitive (adaptive) response (Fig 5). We found that altering the affinities between the sequestrating protein *T* and the enzymes can also implement a similar modulation (S7 Fig). These results show that varying concentration and/or affinities of sequestrating protein can modulate plasticity in response dynamics, even in a simple minimalist system as used here. It is possible that in more complex networks such response modulation can be embedded within the network dynamics, where the activity of the scaffolding protein is allosterically regulated by other proteins or directly by the signal.

## Discussion

Here, we used *in silico* evolution coupled to a biologically realistic rule-based model of proteins to evolve signalling networks displaying ultrasensitive and adaptive responses. Running evolutionary simulations under conditions of enzyme saturation or not, we found that enzyme sequestration (e.g. by scaffolding proteins) is a key network feature enabling such response dynamics. These results from the *in silico* evolution allowed us to design a simple network motif, that featured a scaffolding protein and that can implement both adaptation and ultrasensitivity. The system could be tuned to respond with one of these dynamics by tuning the kinetic parameters and concentrations of the scaffolding protein. These findings highlight that scaffolding proteins, and more generally enzyme sequestration, in natural systems and synthetic biology applications can act as the control point of response dynamics.

A key finding from *in silico* evolved networks is that selection for ultrasensitivity and adaptive response dynamics resulted in the emergence of structural and dynamical patterns featuring enzyme sequestration. This empirical observation indicates that enzyme sequestration is a potential design principle of ultrasensitive and adaptive response dynamics in signalling networks. Indeed, enzyme sequestration is ubiquitous in biological systems where it can emerge from different molecular mechanisms, such as compartmentalisation of proteins [64], inhibitory binding of signalling proteins [50] [53] [32] [33] and scaffolding recruitment [65–69]. Based on these results and this study, we hypothesize that protein sequestration mechanisms contribute to ultrasensitive and/or adaptive response dynamics in nature. Moreover, our results show that it is possible to design and engineer protein sequestration (e.g. through scaffold proteins [70]) to achieve ultrasensitivity and/or adaptive response dynamics.

In natural systems, the activity of scaffold proteins are found to be controlled by transcriptional regulation of their concentration [66] and/or by post-translational modifications and allosteric regulations of their binding affinity to substrates [65]. The findings presented here suggest that such regulation of scaffolding proteins could be directly involved in the tuning of response dynamics of signalling networks where they are found. This suggests that evolution has exploited scaffolding proteins to enable diverse and/or plastic response dynamics at cellular level. Indeed, scaffold proteins are ubiquitously distributed in cellular signalling networks [71] and several experimental studies have shown their involvement in regulating response dynamics [65,66,68,69]. Additionally, we note that the kinase and phosphatase sequestration we describe here is similar to bifunctional enzymes mediating robust homeostatic dynamics as identified in several biological systems [18,72].

From a synthetic biology perspective, the presented findings provide key insights on how altering scaffolding proteins can directly alter response dynamics. Manipulation of allosteric properties and/or concentrations of scaffolding proteins in the MAPK signalling pathways is already shown to result in diverse response dynamics [73–75]. Our findings elucidate the dynamical basis of these observations and provide further guidance to these synthetic approaches. For example, it is increasingly possible to induce or change interaction of enzymes with scaffolding proteins through alteration of common interaction domains [76,77], which could allow introduction of new sequestering interactions in specific systems. These experimental methods, when combined with the theoretical insights presented here can lead to scaffold proteins becoming a key engineering tool for directing and manipulating signalling dynamics [70].

This study, as well as similar studies [14,39], show that *in silico* evolution is a useful approach to discover biochemical principles that are not readily identified in experimental model systems or through analysis of conserved structural features. The ability of evolutionary simulations to provide sample systems implementing a specific function enables the generation of hypotheses that can be subsequently tested in experiments or verified using minimalistic models, as we have done here. Thus, evolution *in silico* can provide us with insights on biochemical features that natural evolution has so successfully exploited. These features can act as *evolutionary* design principles that can further our ability to engineer *de novo* biological systems and understand the natural ones.

## Materials and Methods

### Evolutionary simulations

We used an *in silico* evolution platform, called BioJazz (http://oss-lab.github.io/biojazz/), that combines rule-based models of proteins and their interactions with an evolutionary algorithm [41]. This framework models proteins through their domain structure and allosteric regulation [41,78], properties which are known to be important in signalling networks [65,67,79] [1]. The evolutionary simulations are achieved in BioJazz by encoding rule based model structures in a form of a binary string, which is then modified through specific operators representing most of the biologically plausible mutations mechanisms; protein duplication and deletion, domain duplication and deletion, domain shuffling, genome rearrangement, gene transfer, and point mutation (affecting kinetic rates). The rates for these operators (i.e. the mutation rates for specific mutation types) are controllable by the user through a configuration file (S1 Data). The resulting evolutionary simulations allow theoretically unbounded network complexity, where proteins featuring multiple binding and catalytic domains can emerge [41]. We started evolutionary simulations with three “seed” networks with different structures as shown in Fig 1 and discussed in the main text. For each “seed” network, we run two groups of simulations with different total concentrations of signalling and output proteins, mimicking initial presence of enzyme saturation or not. Under each condition (and seed structure) we have run 10 independent evolutionary simulations.

An evolutionary algorithm implements the iterative process of mutation and selection using a predefined fitness function (see next section). To simulate evolutionary dynamics, we assumed a low mutation rate—high population size regime as explained in [41]. In such a regime, evolution is expected to proceed akin to a random walk, where only fitter mutants are expected to fix in the population and form the basis for next mutants [41]. Thus, we simulate only a single network, from which we generate mutants and replace the resident network based on the probability of fixation calculated from the fitness difference between mutants and the resident genotype as derived by Kimura [80]. The additional parameters controlling evolutionary simulations, such as mutation rates and the allowed size of protein complexes are summarised in a configuration file, which is provided as S1 Data. All presented simulations are run with the same parameters given in this file. The details of all parameters used in evolutionary simulations and encoding of rule-based models are further explained in the BioJazz research paper [41] and online manual: http://oss-lab.github.io/biojazz/.

### Evolved networks

All evolved network models are simulated using mass action kinetics, as implemented originally in ANC and BioJazz [41,78]. The evolved networks are shown in cartoon form and discussed in the main text and their properties summarised in S1 Table. In addition, we provide all network models from the end point of each evolutionary simulation in MATLAB and additional formats (S5 Data). For one sample evolutionary simulation, we provide models from each “generation” of the simulation (S6 Data) as well as models of starting networks (S7 Data). The provided MATLAB files are derived from rule-based models based on mass-action kinetics. The original rule-based models are also provided in the “.mod” format, which can be executed by the rule-based modelling framework Allosteric Network Compiler (ANC) (http://github.com/OSS-Lab/anc) [78], and the “.eqn” format, which can be executed by the MATLAB compatible mathematical analysis platform Facile (http://github.com/OSS-Lab/facile) [81].

### Formalisation of and selection for adaptive and ultrasensitive dynamics

#### Ultrasensitivity

We define ultrasensitivity as done before [16,82]: a nonlinear dose-response function where a small change in the input is amplified into a large change in steady state response at a distinct input level (S1A Fig). In order to quantify the ultrasensitive dynamics in simulated networks, we use a previously defined fitness function [41] and as shown in S1 Fig. In brief, we implement three steps of input signals with both ramping-up and ramping-down functions. After each perturbation on input signals, we record the steady-state level of the output response. Then we calculate the response of the system for each of these perturbations as:

$$\begin{array}{ccccc} \Delta y_{i+} & = & y_{i{+}_{max}} & - & y_{i{+}_{min}}\\ \Delta y_{i-} & = & y_{i{-}_{max}} & - & y_{i{-}_{min}} \end{array},$$ (1)

where the ‘+/-’ notation indicates response to ramping-up and ramping-down perturbations respectively. Using these measurements we calculate *S*_*amp*_, as the amplitude of the response to the second perturbation, and *S*_*ult*_, the sensitivity at the threshold as the ratio between responses to the second perturbation and to the first/third perturbations:

$$\begin{array}{ccc} S_{amp} & = & \frac{(\Delta y_{2+}+\Delta y_{2-})/2}{y_{total}}\\ S_{ult} & = & \sqrt{\left(\frac{s_{u1}}{r_{u}+S_{u1}})\cdot (\frac{s_{u3}}{r_{u}+S_{u3}}\right)} \end{array},$$ (2)

where *y*_*total*_ is the maximum possible response and *r*_*u*_ is a scaling factor, *s*_*u*1_ = (Δ*y*_2+_+Δ*y*_2−_)/(Δ*y*_1+_+Δ*y*_1−_) and *s*_*u*3_ = (Δ*y*_2+_+Δ*y*_2−_)/(Δ*y*_3+_+Δ*y*_3−_). The final ultrasensitivity score *F* is:

$$F=\sqrt{S_{amp}\cdot S_{ult}}.$$ (3)

#### Adaptive response dynamics

Biochemical adaptation refers to the function that many signaling systems return to their pre-stimulated state after responding to a sustained stimulus (S1B Fig). A mathematical description for adaptation can be quantified by two terms: adaptation sensitivity (*A*_sens_) to the input perturbation and adaptation precision (*A*_prec_) [30,83]. The sensitivity is defined as the maximum of response to the sustained input stimulus (S1B Fig) and can be calculated as:

$$A_{sens}=|\frac{(O_{*}-O_{0})/O_{0}}{(I_{1}-I_{0})/I_{0}}|,$$ (4)

while the adaptation precision can be calculated as:

$$A_{prec}={\left|\frac{(O_{1}-O_{0})/O_{0}}{(I_{1}-I_{0})/I_{0}}\right|}^{-1}.$$ (5)

For both equations, *O*_0_ and *O*_1_ indicate the pre- and post-stimuli response levels, while *I*_0_ and *I*_1_ indicate the corresponding input (i.e. signal) level. The term O_*_ indicates the peak response produced as shown in S1B Fig. The “perfect adaptation” emerges if output response returns exactly to the pre-stimulated state (*O*_1_ = *O*_0_).

The fitness function for adaptive response dynamics is based on a previously published function [41]. We calculate both maximum response to input perturbations ($\Delta O_{i}^{max+/-}\Delta O_{i}^{max+/-}$) and adaptive precision (i.e. difference between pre- and post-perturbation responses, $\Delta O_{i}^{ss+/-}$). For each square pulse signal perturbation, we calculate the “level of adaptive response” as $w_{i}=\sqrt{\frac{\Delta O_{i}^{max+}}{C}\cdot \frac{K}{K+\Delta O_{i}^{ss+}}}\cdot \sqrt{\frac{\Delta O_{i}^{max-}}{C}\cdot \frac{K}{K+\Delta O_{i}^{ss-}}}$, where *C* is a normalisation factor to scale $\Delta O_{i}^{max+/-}$ and $\Delta O_{i}^{ss+/-}$ to the interval [0,1], and *K* is a threshold parameter forcing a minimal response level (S1D Fig). The final adaptive response fitness score is calculated as geometric mean of scores to a series of perturbation steps of different magnitude $w=\sqrt[{n+1}]{\Pi_{i=0}^{n}w_{i}}$. We have used three distinct step-signals with different magnitudes (i.e. 1, 10, 100) (see S1D Fig).

### Model for a signalling cycle motif featuring enzyme sequestration

We use the well-known phosphorylation—dephosphorylation cycle seen in natural signalling networks and extend it with a sequestering protein to form a minimal signalling motif. In this motif, a sequestrating protein (*T*) can bind both the kinase (*K*) and the phosphatase (*P*), thus making these enzymes inaccessible to their substrate (*S* and *S*_*p*_). We developed a generic model of this system that consists of 10 reaction rate constants and 9 chemical species (Fig 4A). We analysed the dynamics of this system by writing ordinary differential equations that describe the change in concentration of each of the 9 species (S2–S4 Data). In order to explore the different response dynamics of this generic model, we sampled parameter sets from a biologically feasible range (see Table 1). We used the same fitness functions as in the evolutionary simulations to quantify the nature of the response of this signalling motif to an incoming signal as ultrasensitive or adaptive. The signal presence is simulated as changes in the kinase concentration level. To explore effects of enzyme saturation, we sampled the generic model at two conditions: enzyme saturated ([*P*_*tot*_] = 0.1, [*S*_*tot*_] = 1) and enzyme unsaturated ([*P*_*tot*_] = 0.1, [*S*_*tot*_] = 0.1). A computer executable for this model is provided as MATLAB, Mathematica and PDF files (S2, S3 and S4 Data).

## Supporting Information

S1 FigFormalisation and quantification of ultrasensitive and adaptive response dynamics.(A) The ultrasensitive response dynamics. The plot is showing the steady state levels of input signal and output response at steady state. Axis labeled with *I* and *O* represent the input level and output response respectively. (B) The adaptive response dynamics. The plots show the temporal dynamics with *x*-axis labeled with *t* representing time, *I*_0_ and *O*_0_ represent pre-stimulus level of input signal and output response, *I*_1_ and *O*_1_ represent respective levels after stimulus, O_*_ represents the level of output response with largest deviation from its pre-stimulus level. **(C)** Sample response dynamics describing the measures for calculating the ultrasensitivity fitness function. Each ramp-up and ramp-down of the signal (blue) is introduced after the system response (green) reaches steady state. The differences in steady state output between different signal levels, indicated as Δ*y* values on the plot, are used to calculate the amplitude and ultrasensitivity scores (see Methods). **(D)** Sample input (blue)–output (green) response dynamics describing the measures for calculating the adaptive response fitness function (see Methods). The parameters in adaptive fitness function, measuring initial response level ($\Delta O_{i}^{max+/-}$) and adaptation precision ($\Delta O_{i}^{ss+/-}$), are shown.(TIFF)

S2 Fig Structure of all evolved ultrasensitive networks, along with the simulation identification number. The networks are grouped according to the starting concentrations of signalling proteins and output protein (initial conditions), and the starting network structures as shown in Fig 1. The information presented on the network cartoons is the same as in Fig 1. Note that many evolved networks feature isolated proteins that evolved from duplications and mutations.
(TIFF)

S3 Fig Average level of enzyme sequestration by proteins other than the output protein (*E-T complexes*) and by the output protein, i.e. substrate (*E-S complexes*), in all evolved ultrasensitive networks. The kinases and phosphatases are shown as orange and the green dots respectively, while the numbers denote the network number as shown in S2 Fig. The cases where we have two dots with the same colour and number indicate the allosteric forms of a given enzyme. The different panels show the results from simulations with different initial conditions and the starting network structures, as shown in Fig 1. The dashed lines indicate full complex formation of the enzymes with no free form available.
(EPS)

S4 Fig Parameter sampling in the minimal signalling cycle motif. For a given parameter set (shown as a black dot), the x-axis shows the adaptive response dynamics score and the y-axis shows the ultrasensitivity score (see text and S1 Fig for response dynamics scores). (A) Sampled network parameters under substrate-saturating condition ([*S*_*total*_] = 1 and [*P*_*total*_] = 0.1). (B) Sampled network parameters under non-saturating condition ([*S*_*total*_] = 0.1 and [*P*_*total*_] = 0.1). On both panels, the most adaptive and ultrasensitive networks are shown on top-left and bottom-right corner respectively (data point indicated with an arrow). For the adaptive dynamics, the signal (blue) and phosphorylated substrate fraction (green) are shown against time (x-axis). For the ultrasensitive dynamics, the steady state phosphorylated substrate fraction is shown against signal (concentration of the kinase, x-axis).
(EPS)

S5 Fig Parameter regimes for ultrasensitivity and ensuing response dynamics. (A) Distribution of key parameters in the sampled parameter sets that have resulted in an ultrasensitive score above 0.8. The x- and y-axes show the Michaelis-Menten constants for the kinase (*K*) and phosphatase (*P*) respectively (*K*_*M*,*K*_ = (*k*_2_+*k*_3_)/*k*_1_, *K*_*M*,*P*_ = (*k*_5_+*k*_6_)/*k*_4_). There are two separate regimes with differing *K*_*M*, *P*_ values, which also differ in the sequestering protein—enzyme binding affinities; see insets showing sequestering protein—kinase (*K*_*D*,*K*_ = *k*_8_/*k*_7_) and sequestering protein—phosphatase (*K*_*D*,*P*_ = *k*_10_/*k*_9_) binding coefficients on the x- and y-axis respectively. (B) Response dynamics in the parameter regime, where with low *K*_*M*, *P*_ and high *K*_*D*, *P*_. The panel shows the steady state concentrations of the different species against the input level (x-axis). (C) Response dynamics in the parameter regime, where with high *K*_*M*, *P*_ and low *K*_*D*, *P*_. The panel shows the steady state concentrations of the different species against the input level (x-axis).
(EPS)

S6 Fig Distribution of key parameters in the sampled parameter sets that have resulted in high ultrasensitive (> 0.8, top row) and adaptive scores (>0.3, bottom row), when assuming an enzyme-non-saturated regime ([*S*_*total*_] = 0.1, [*P*_*total*_] = 0.1). Panels on the left show the distribution of Michaelis-Menten constants, for kinase: *K*_*M*,*K*_ = (*k*_2_+*k*_3_)/*k*_1_ (*x*-axis) and phosphatase *K*_*M*,*P*_ = (*k*_5_+*k*_6_)/*k*_4_ (*y*-axis). Panels on the right show the distribution of affinities of sequestrating protein *T* with kinase and phosphatase: *K*_*D*,*K*_ = *k*_8_/*k*_7_ and *K*_*D*,*P*_ = *k*_10_/*k*_9_. Note that all four panels are plotted on the same logarithmic range. Each black dot represents a parameter set and the colours shows density of parameters.
(TIFF)

S7 Fig Sampling of affinity parameters (*k_7_*, *k_8_*, *k_9_*, *k_10_*) in a system with a given parameter set. The ultrasensitive and adaptive scores are shown on the x- and y-axis respectively.
(EPS)

S1 Table Complexity of evolved networks in terms of numbers of species, reactions, proteins, domains, interactions. (DOCX)

S1 Data A configuration file for BioJazz, listing all simulation parameters. (CFG)

S2 Data Matlab code describing the minimal signalling cycle model. (ZIP)

S3 Data A Mathematica notebook for simulating the minimal signalling cycle model. (NB)

S4 Data A PDF file generated from Mathematica notebook describing the minimal signalling cycle model. (PDF)

S5 Data Models for all evolved adaptive and ultrasensitive networks. (ZIP)

S6 Data Models of all generations in evolutionary simulation No. 13. (ZIP)

S7 Data Models for all three starting networks. (ZIP)
