# Supplementary figures and images for: Enzyme Sequestration as a Tuning Point in Controlling Response Dynamics of Signalling Networks
Source: PLoS Comput Biol. 2016 May 10;12(5):e1004918. doi: 10.1371/journal.pcbi.1004918 (PMC4862689; doi:10.1371/journal.pcbi.1004918)

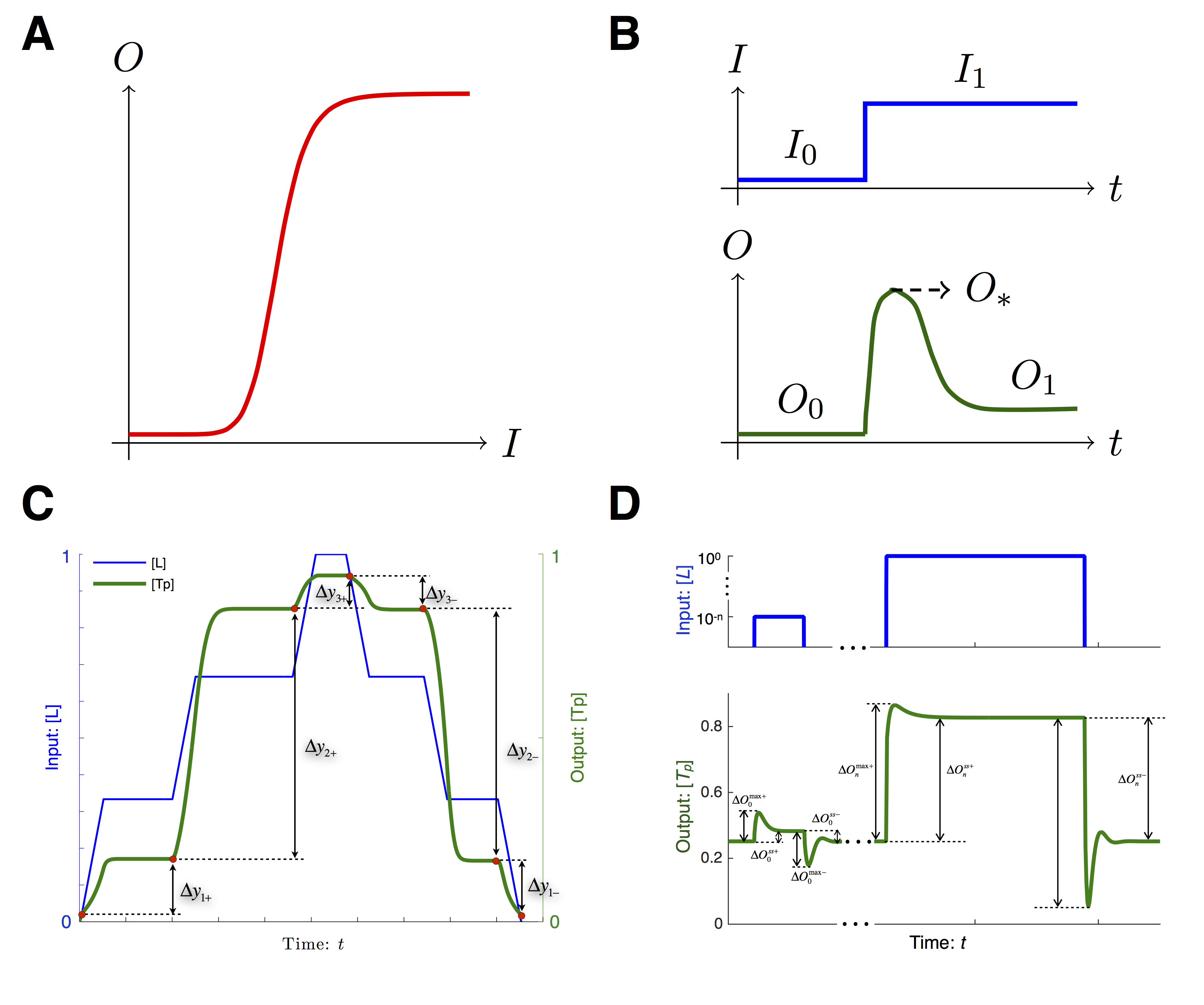

Supplement: S1 Fig — (A) The ultrasensitive response dynamics. The plot is showing the steady state levels of input signal and output response at steady state. Axis labeled with I and O represent the input level and output response respectively. (B) The adaptive response dynamics. The plots show the temporal dynamics with x-axis labeled with t representing time, I0 and O0 represent pre-stimulus level of input signal and output response, I1 and O1 represent respective levels after stimulus, O* represents the level of output response with largest deviation from its pre-stimulus level. (C) Sample response dynamics describing the measures for calculating the ultrasensitivity fitness function. Each ramp-up and ramp-down of the signal (blue) is introduced after the system response (green) reaches steady state. The differences in steady state output between different signal levels, indicated as Δy values on the plot, are used to calculate the amplitude and ultrasensitivity scores (see Methods). (D) Sample input (blue)–output (green) response dynamics describing the measures for calculating the adaptive response fitness function (see Methods). The parameters in adaptive fitness function, measuring initial response level (ΔOimax+/-) and adaptation precision (ΔOiss+/-), are shown. (TIFF) [file pcbi.1004918.s001.tiff]

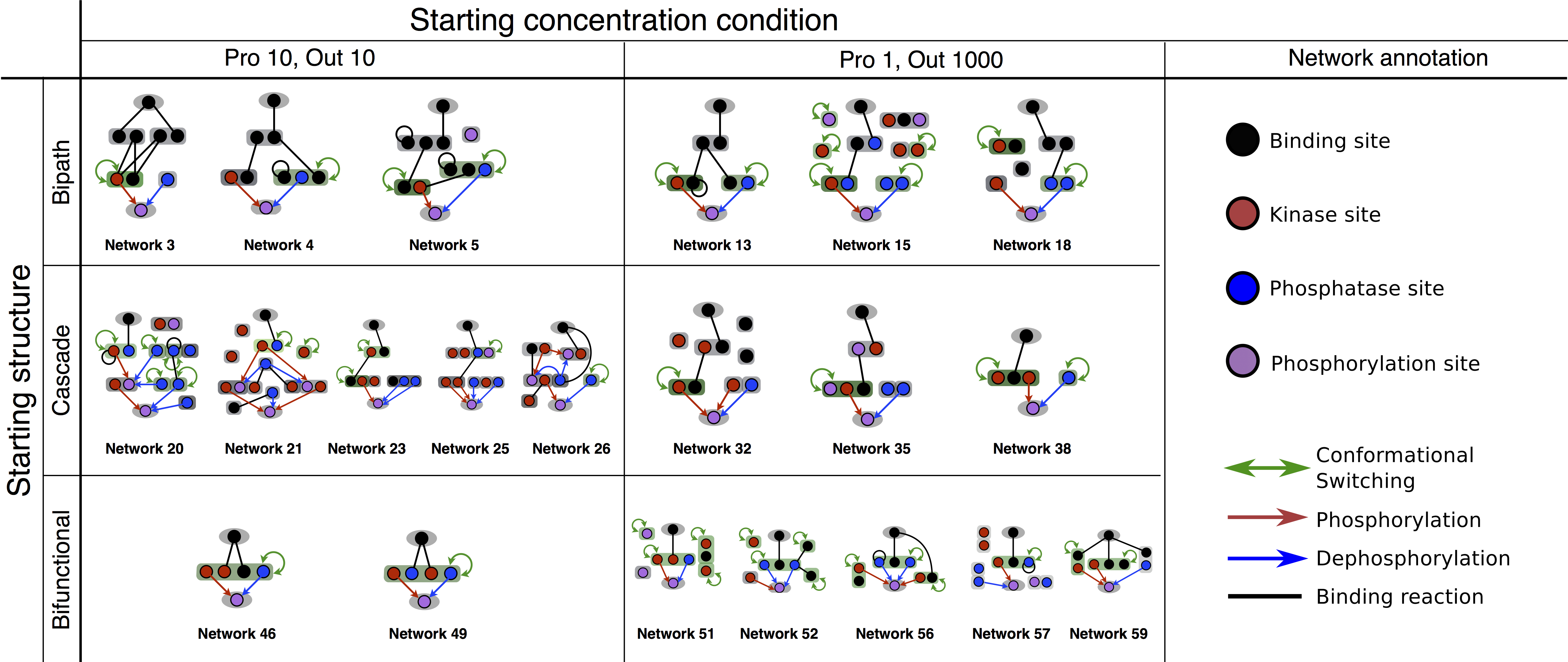

Supplement: S2 Fig — The networks are grouped according to the starting concentrations of signalling proteins and output protein (initial conditions), and the starting network structures as shown in Fig 1. The information presented on the network cartoons is the same as in Fig 1. Note that many evolved networks feature isolated proteins that evolved from duplications and mutations. (TIFF) [file pcbi.1004918.s002.tiff]

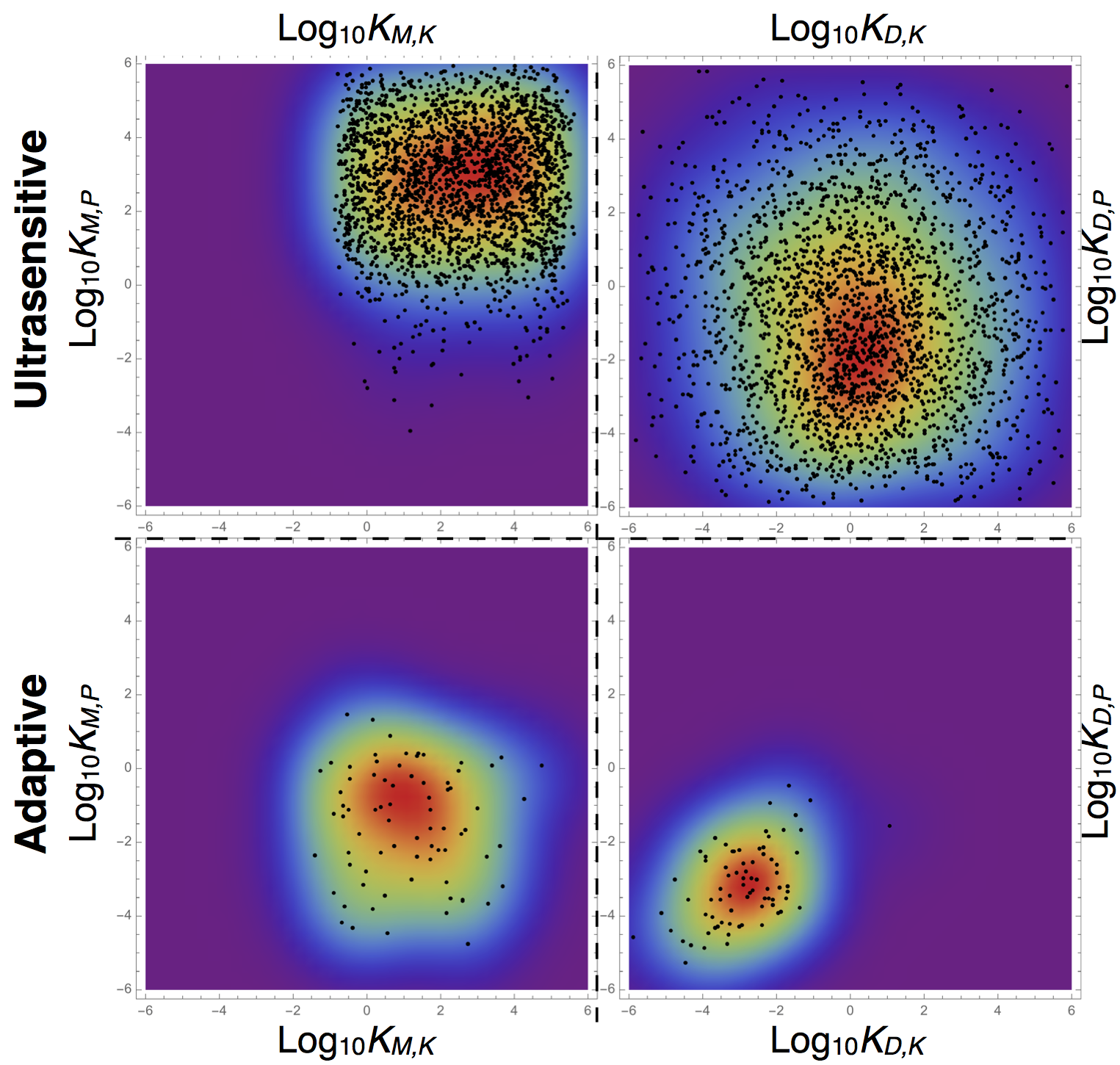

Supplement: S6 Fig — Panels on the left show the distribution of Michaelis-Menten constants, for kinase: KM,K = (k2+k3)/k1 (x-axis) and phosphatase KM,P = (k5+k6)/k4 (y-axis). Panels on the right show the distribution of affinities of sequestrating protein T with kinase and phosphatase: KD,K = k8/k7 and KD,P = k10/k9. Note that all four panels are plotted on the same logarithmic range. Each black dot represents a parameter set and the colours shows density of parameters. (TIFF) [file pcbi.1004918.s006.tiff]
