# Supplementary material for: Enzyme Sequestration as a Tuning Point in Controlling Response Dynamics of Signalling Networks
Source: PLoS Comput Biol. 2016 May 10;12(5):e1004918. doi: 10.1371/journal.pcbi.1004918 (PMC4862689; doi:10.1371/journal.pcbi.1004918)
Supplement: S1 Table — (DOCX) [file pcbi.1004918.s008.docx]

**Title: Enzyme sequestration as a tuning point in controlling response dynamics of signalling networks**

**Authors:** Song Feng^1^, Julien F. Ollivier ^2^, Orkun S. Soyer ^1^*

**Supplementary Figure Legends**

**Table S1. Complexity of evolved ultrasensitive networks and adaptive networks.**

| Network # | Species | Reaction | Protein | Domain | Interaction (rule) |
| --- | --- | --- | --- | --- | --- |
| 3 | 41 | 145 | 6 | 9 | 13 |
| 4 | 31 | 127 | 5 | 9 | 11 |
| 5 | 50 | 249 | 6 | 11 | 19 |
| 13 | 32 | 123 | 5 | 8 | 13 |
| 15 | 19 | 50 | 9 | 15 | 7 |
| 18 | 17 | 43 | 7 | 10 | 6 |
| 20 | 62 | 270 | 8 | 14 | 18 |
| 21 | 47 | 144 | 11 | 16 | 16 |
| 23 | 46 | 216 | 5 | 10 | 12 |
| 25 | 17 | 38 | 5 | 11 | 8 |
| 26 | 62 | 208 | 7 | 11 | 12 |
| 32 | 18 | 46 | 8 | 11 | 7 |
| 35 | 17 | 43 | 5 | 9 | 8 |
| 38 | 15 | 38 | 4 | 6 | 6 |
| 46 | 23 | 88 | 3 | 6 | 8 |
| 49 | 23 | 88 | 3 | 6 | 8 |
| 51 | 15 | 46 | 6 | 10 | 6 |
| 52 | 27 | 81 | 7 | 9 | 8 |
| 56 | 34 | 139 | 5 | 9 | 14 |
| 57 | 24 | 104 | 6 | 11 | 9 |
| 59 | 22 | 59 | 5 | 9 | 9 |
| Adaptive 1 | 23 | 59 | 5 | 9 | 9 |
| Adaptive 2 | 22 | 62 | 6 | 10 | 7 |
| Bipath | 15 | 30 | 5 | 8 | 5 |
| Cascade | 15 | 22 | 6 | 8 | 5 |
| Bifunctional | 9 | 18 | 3 | 5 | 3 |
