# Supplementary material for: Enzyme Sequestration as a Tuning Point in Controlling Response Dynamics of Signalling Networks
Source: PLoS Comput Biol. 2016 May 10;12(5):e1004918. doi: 10.1371/journal.pcbi.1004918 (PMC4862689; doi:10.1371/journal.pcbi.1004918)

Scaffold protein titration motif

The model description

This particular motif describe one phosphorylation-desphosphorylation cycle (can be generalized to any futile cycles) with both kinase (K) and phosphatase (P) can be titrated by a scaffold protein (T).

$\mathrm{K} + \mathrm{S} \rightleftharpoons \mathrm{KS} \rightarrow \mathrm{K} + S_p$
$\mathrm{P} + S_p \rightleftharpoons \mathrm{PS}_p \rightarrow \mathrm{P} + \mathrm{S}$
$\mathrm{T} + \mathrm{K} \rightleftharpoons \mathrm{TK}$
$\mathrm{T} + \mathrm{P} \rightleftharpoons \mathrm{TP}$
$\varnothing \rightarrow \mathrm{K}$
$\mathrm{K} \rightarrow \varnothing$

The above reactions show a simple system that composed of one scaffold protein, one kinase, one phosphatase and one substrate. Here we try to descibe this simple system with differential equation following the mass action kinet-
ics.

$$\frac{d[K]}{dt} = -k[1][K][S] + k[2][\mathrm{KS}] + k[3][\mathrm{KS}] - k[7][T][K] + k[8][\mathrm{TK}] + k[11]\, k_d - k_d[K],$$
$$\frac{d[P]}{dt} = -k[4][P][S_p] + k[5][\mathrm{PS}_p] + k[6][\mathrm{PS}_p] - k[9][T][P] + k[10][\mathrm{TP}],$$
$$\frac{d[S]}{dt} = -k[1][K][S] + k[2][\mathrm{KS}] + k[6][\mathrm{PS}_p],$$
$$\frac{d[S_p]}{dt} = -k[4][P][S_p] + k[3][\mathrm{KS}] + k[5][\mathrm{PS}_p],$$
$$\frac{d[\mathrm{KS}]}{dt} = k[1][K][S] - k[2][\mathrm{KS}] - k[3][\mathrm{KS}],$$
$$\frac{d[\mathrm{PS}_p]}{dt} = k[4][P][S_p] - k[5][\mathrm{PS}_p] - k[6][\mathrm{PS}_p],$$
$$\frac{d[T]}{dt} = -k[7][T][K] + k[8][\mathrm{TK}] - k[9][T][P] + k[10][\mathrm{TP}]\,,$$
$$\frac{d[\mathrm{TK}]}{dt} = k[7][T][K] - k[8][\mathrm{TK}],$$
$$\frac{d[\mathrm{TP}]}{dt} = k[9][T][P] - k[10][\mathrm{TP}].$$

And the system need to follow these conservation equations:

$[K] + [\mathrm{KS}] + [\mathrm{TK}] = [K_{\mathrm{tot}}],$
$[P] + [\mathrm{PS}_p] + [\mathrm{TP}] = [P_{\mathrm{tot}}],$
$[S] + [S_p] + [\mathrm{KS}] + [\mathrm{PS}_p] = [S_{\mathrm{tot}}],$
$[T] + [\mathrm{TK}] + [\mathrm{TP}] = [T_{\mathrm{tot}}].$

In the following setion, we will solve the differential equations to understand the dynamics and behaviour of such system.

Understanding the dynamics of the simple system with input pertubations (numerical study)

Since, it is a bit difficult to solve the differential equations analytically. Here we try to study them numerically. By defining two different way to characterising the dynamics with scoring their tempral dynamics when presented with input signal perturbation (the changing of [T]). The quantification can be derived from the actually fitness funcitons for ultrasensitive response and adaptive response. Then we save all the parameter sets as well as their score on ultrasensitivity and adaptation.

```
Clear["Global`*"];
SetDirectory[NotebookDirectory[]];
kd = 10;
des = {-k[1] * x[1][t] * x[3][t] + k[2] * x[5][t] + k[3] * x[5][t] -
     k[7] * x[1][t] * x[7][t] + k[8] * x[8][t] + k11[t] * kd - kd * x[1][t],
    -k[4] * x[2][t] * x[4][t] + k[5] * x[6][t] + k[6] * x[6][t] -
     k[9] * x[2][t] * x[7][t] + k[10] * x[9][t],
    -k[1] * x[1][t] * x[3][t] + k[2] * x[5][t] + k[6] * x[6][t],
    -k[4] * x[2][t] * x[4][t] + k[3] * x[5][t] + k[5] * x[6][t],
    k[1] * x[1][t] * x[3][t] - k[2] * x[5][t] - k[3] * x[5][t],
    k[4] * x[2][t] * x[4][t] - k[5] * x[6][t] - k[6] * x[6][t],
    -k[7] * x[1][t] * x[7][t] - k[9] * x[2][t] * x[7][t] +
     k[8] * x[8][t] + k[10] * x[9][t],
    k[7] * x[1][t] * x[7][t] - k[8] * x[8][t],
    k[9] * x[2][t] * x[7][t] - k[10] * x[9][t], 0};

init = {totK, totP, totS, 0, 0, 0, totT, 0, 0, 1. * 10^-4};
(*init={tot[1],tot[2],tot[3],0.00001,0.00001,0.00001,totT,0.00001,0.00001};*)

AbsoluteTiming[
 totK = 0.0001; totP = 0.1; totS = 1;
 stepNum = 5;
 sampleSize = 100000;

 pars = {};
 vars = Array[x, 9]; AppendTo[vars, k11];
dvars = Thread[Derivative[1][vars]];
 SeedRandom[IntegerPart[SessionTime[]]];
 ts = {};
 For[num = 1, num ≤ sampleSize, num++,
  Block[{k, T, ssthreshold}, k[n_] := k[n] = 10^(RandomReal[] * 6 - 3);
    (*tot[n_]:=tot[n]=10^(RandomReal[]*4-3);*)
    (*ksTest1=Array[k,10];*)
    (*totT=1.*^-3;*)
    totT = 1. * 10^(RandomReal[] * 4 - 3);

    Block[{tPer, step},
     step = 0;
     tPer = {};
     ssthreshold = 1.*^-5;
     (* Print[des]; *){sol} = NDSolve[{Through[dvars[t]] == des,
        Through[vars[0]] == init, With[{df = Through[dvars[t]]},
         WhenEvent[Norm[df] < ssthreshold, {AppendTo[tPer, t], step = step + 1,
```

```
          If[step > stepNum, "StopIntegration"], k11[t] → 10 * k11[t]}]]},
       vars, {t, 0, 200 000}, MaxSteps → 10 000];
     ts = tPer;
     If[Length[ts] == stepNum + 1 && AllTrue[ts, Positive],
      x4 = Evaluate[x[4][ts - 0.001] /. sol];
      xT = Evaluate[(x[7][ts - 0.001] + x[8][ts - 0.001] + x[9][ts - 0.001]) /. sol];

      us = Sqrt[((Abs[(x4[[4]] - x4[[3]])] / totS) *
          Min[((Abs[(x4[[4]] - x4[[3]])] / Max[Abs[(x4[[3]] - x4[[1]])], 0.001] +
                Abs[(x4[[4]] - x4[[3]])] / Max[
                  Abs[(x4[[stepNum + 1]] - x4[[4]])], 0.001]) / 2) / 10.0, 1.0])];

      ad = 0.0001;
      For[i = 1, i ≤ stepNum, i++,
       ad = ad * Sqrt[
          (Min[(Max[Abs[Evaluate[x[4][Range[ts[[i]], ts[[i + 1]], 1]] /. sol] -
                   Evaluate[x[4][ts[[i]]] /. sol]]] / (0.2 * totS)), 1.0] *
            ((0.01) / (Max[Abs[(x4[[i + 1]] - x4[[i]]) / totS], 0.01])))];
      ];
      ad = (ad / 0.0001) ^ (1 / (stepNum));

      ks = Array[k, 10];
      AppendTo[pars, Join[ks, {totT, totK, totP, totS, us, ad, num,
          (ks[[2]] + ks[[3]]) / ks[[1]], (ks[[5]] + ks[[6]]) / ks[[4]], ks[[8]] / ks[[7]], ks[[10]] / ks[[9]]}]];
     ];
    ];
   ];
  ];
 ]

(*Plot@@{{{(x[7][t]+x[8][t]+x[9][t]),x[4][t]}/.sol},
   Flatten@{t,x[1]["Domain"]/.sol},PlotLegends→{"Ttot","Sp"}}
 ListPlot[Transpose@{xT,x4},PlotRange→{0,10}]*)
(*Print[pars];*)
transPars = Transpose[pars];
Export["saturationSampling.csv", transPars];
(*Export["unsaturationSampling.csv",transPars];*)

ListPlot[Transpose[{transPars[[15]], transPars[[16]]}],
 PlotRange → {{0, 1}, {0, 1}},
 (*AxesLabel→{"Ultrasensitive score","Adaptive score"},*)
 Ticks → {{0, 0.5, 1}, {0.5, 1}}, PlotStyle → {Thick, PointSize[0.01]},
 PlotTheme → "Monochrome", PlotLabel → None,
 LabelStyle → {24, GrayLevel[0]}, ImageSize → Large]
```

Optional: study on the sampled parameters

```
maxAndIndex[a_] :=
 {#, First@SparseArray[UnitStep[a - #]]["AdjacencyLists"]} &@Max@a

maxAndIndex[transPars[[15]]]

{0.948424, 40 183}
```

```
maxAndIndex[transPars[[16]]]
```

```
{0.581416, 46757}
```

```
usIndex = maxAndIndex[transPars[[15]]] // Last;
adIndex = maxAndIndex[transPars[[16]]] // Last;
pars[[usIndex]]
```

```
{0.0152058, 550.394, 26.7347, 0.0255653, 120.706, 0.576381,
 60.5834, 41.0397, 746.636, 0.00360504, 8.25277, 0.0001, 0.1, 1,
 0.948424, 0.0566003, 40686, 37954.4, 4744.02, 0.677409, 4.82838 × 10^-6}
```

```
pars[[adIndex]]
```

```
{29.7712, 0.00985813, 2.01657, 111.111, 8.04903, 436.845, 146.073,
 0.00268851, 48.0433, 0.00798703, 0.851575, 0.0001, 0.1, 1, 0.0182966,
 0.581416, 47351, 0.0680666, 4.00404, 0.0000184052, 0.000166246}
```

```
init = {totK, totP, totS, 0, 0, 0, totT, 0, 0, 1. * 10^-4};
maxAndIndex[a_] :=
 {#, First@SparseArray[UnitStep[a - #]]["AdjacencyLists"]} &@Max@a
usIndex = maxAndIndex[transPars[[15]]] // Last;
adIndex = maxAndIndex[transPars[[16]]] // Last;
stepNum = 5;
maxPars = Solve[Array[k, 10] == pars[[usIndex]][[Range[10]]]];
totT = pars[[usIndex]][[11]];
Block[{tPer, step},
  step = 0;
  tPer = {};
  ssthreshold = 1.*^-5;
  (* Print[des]; *)
  {sol} = NDSolve[{Through[dvars[t]] == des, Through[vars[0]] == init,
      With[{df = Through[dvars[t]]},
       WhenEvent[(Norm[df] < ssthreshold), {AppendTo[tPer, t], step = step + 1,
         If[step > stepNum, "StopIntegration"], k11[t] → 10 * k11[t]}]]} /.
     maxPars, vars, {t, 0, 200000}, MaxSteps → 10000];
  ts = tPer;
  x4 = Evaluate[x[4][ts - 0.001] /. sol] / totS;
  k11t = Evaluate[(k11[ts - 0.001]) /. sol];
 ];
fittedHill = FindFit[Transpose@{k11t, x4},
  a + (b - a) * hillK / (hillK + x^(-n)), {a, b, hillK, n}, x]
Show[LogLinearPlot[a + (b - a) * hillK / (hillK + x^(-n)) /. fittedHill,
  {x, 10^-4, 10}, (*Ticks→{Automatic,{0,0.5,1}},*) PlotRange → {-0.05, 1.05},
  AxesLabel → {"[T]", "[Sp]/[Stot]"}, PlotTheme → "Monochrome",
  PlotStyle → {Thickness[0.01]}], ListLogLinearPlot[Transpose@{k11t, x4},
  PlotTheme → "Monochrome", PlotMarkers → {Automatic, 24}],
 PlotLabel → None, LabelStyle → {24, GrayLevel[0]}, ImageSize → Large]
{a → -0.0000412076, b → 0.99448, hillK → 379521., n → 4.60234}
```

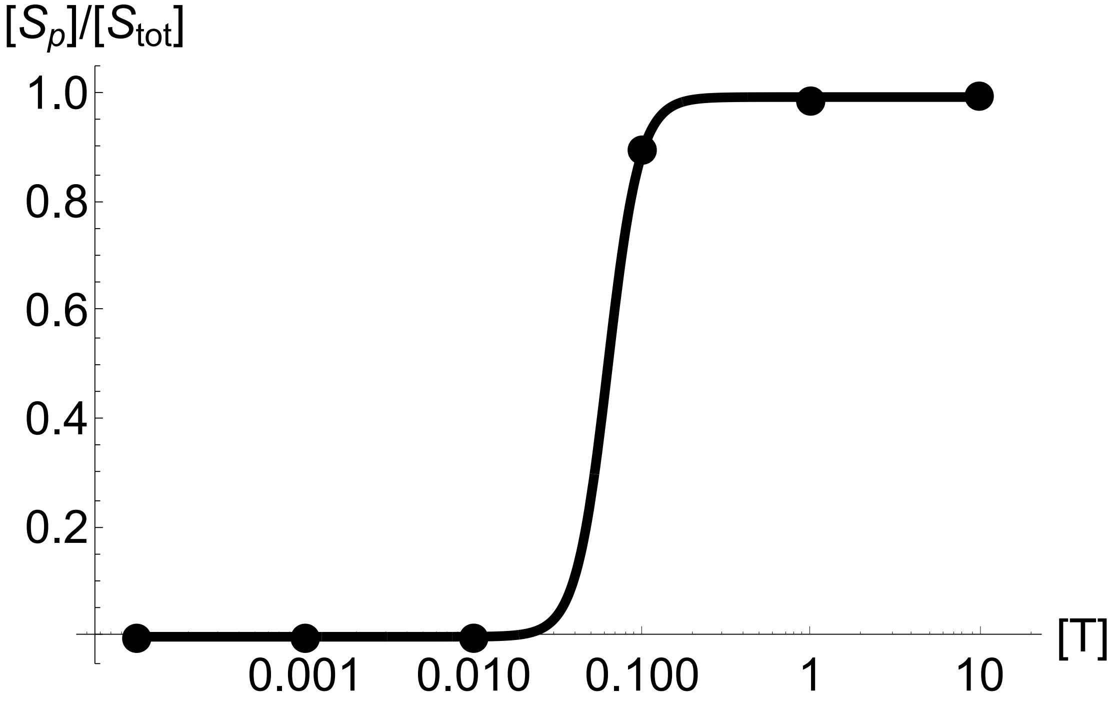

```
init = {totK, totP, totS, 0, 0, 0, totT, 0, 0, 0.01};
stepNum = 3;
maxPars = Solve[Array[k, 10] == pars[[adIndex]][[Range[10]]]];
totT = pars[[adIndex]][[11]];
Block[{tPer, step},
  step = 0;
  tPer = {};
  ssthreshold = 1.*^-5;
  (* Print[des]; *)
  {sol} = NDSolve[{Through[dvars[t]] == des, Through[vars[0]] == init,
      With[{df = Through[dvars[t]]},
       WhenEvent[Norm[df] < ssthreshold, {AppendTo[tPer, t], step = step + 1,
          If[step > stepNum, "StopIntegration"], k11[t] → 5 * k11[t]}]]} /.
     maxPars, vars, {t, 0, 200 000}, MaxSteps → 10 000];
  ts = tPer;
  x4 = Evaluate[x[4][ts - 0.001] /. sol];
  k11t = Evaluate[(k11[ts - 0.001]) /. sol];
 ];

input = LogPlot[{{k11[t]} /. sol}, {t, 0, ts[[stepNum + 1]] - 0.01},
   PlotLegends → Placed[{"[K]"}, {0.15, 0.95}], PlotTheme → "Monochrome",
   PlotStyle → {Blue, Dashed, Thickness[0.007]}, Ticks → {},
   LabelStyle → {18, GrayLevel[0]}, ImageSize → Large,
   ImagePadding → 50, Frame → {True, True, False, False},
   FrameStyle → {Automatic, Blue, Automatic, Automatic}];

actualInput = LogPlot[{{x[1][t]} /. sol}, {t, 0, ts[[stepNum + 1]] - 0.01},
   PlotLegends → Placed[{"[K]"}, {0.15, 0.95}], PlotTheme → "Monochrome",
   PlotStyle → {Blue, Dashed, Thickness[0.007]}, Ticks → {},
   LabelStyle → {18, GrayLevel[0]}, ImageSize → Large,
   ImagePadding → 50, Frame → {True, True, False, False},
   FrameStyle → {Automatic, Blue, Automatic, Automatic}];

output = Plot[{{x[4][t] / totS} /. sol}, {t, 0, ts[[stepNum + 1]] - 0.01},
   PlotLegends → Placed[{"[Sp]/[Stot]"}, {0.45, 0.95}], PlotRange → {0, 1},
   PlotStyle → {Darker[Green], Thickness[0.01]}, Ticks → {0, 0.5, 1},
   LabelStyle → {18, GrayLevel[0]}, ImageSize → Large, ImagePadding → 50,
   (*Axes→False,*)Frame → {False, False, False, True},
   FrameTicks → {None, None, None, {0, 0.5, 1}},
   FrameStyle → {Automatic, Automatic, Automatic, Darker[Green]}];
```

```
adPlot = Overlay[{output, input}]
Export["scaffoldTitrationVaringKAd.eps", adPlot];
Export["scaffoldTitrationVaringKAd.pdf", adPlot];
```

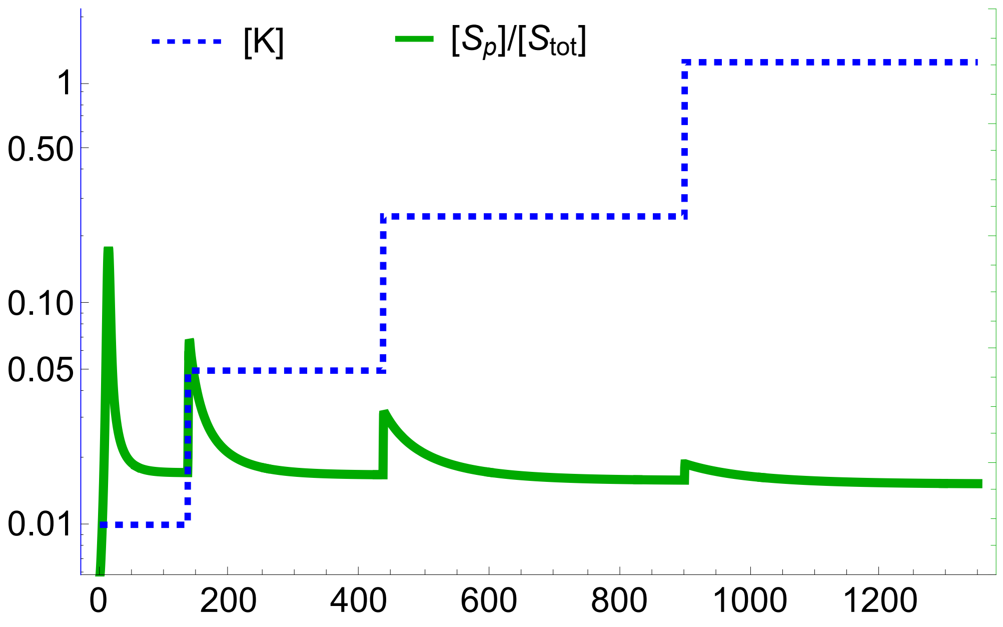

Supplement: S4 Data — (PDF) [file pcbi.1004918.s012.pdf]
